# “We are a club none of us wanted to join”: exploring brain tumor online discussion forum content

**DOI:** 10.1007/s00520-025-09545-z

**Published:** 2025-05-28

**Authors:** Christy Muasher-Kerwin, M. Courtney Hughes, Michelle L. Foster, Samantha M. Econie

**Affiliations:** 1 Department of Physical Therapy, Northern Illinois University, DeKalb, IL 60115 USA; 2 Department of Public Health, Northern Illinois University, DeKalb, IL USA; 3 Department of Nutrition and Dietetics, Northern Illinois University, DeKalb, IL USA

**Keywords:** Online forum, Brain tumor, Caregivers, Peer support, Applied thematic analysis

## Abstract

**Purpose:**

Living with a brain tumor or caring for someone affected by one presents significant emotional, physical, and financial challenges. Patients often contend with difficult treatment decisions and symptoms that disrupt daily life, while informal caregivers must navigate complex medical needs, cognitive and behavioral changes, and the psychological toll of supporting a loved one through a life-altering diagnosis. This study aims to explore the content of brain tumor online discussion forums to learn more about the experiences, concerns, and coping strategies shared by brain tumor patients and their informal caregivers.

**Methods:**

This study collected data from persons who posted on four online forums in September 2024. The analysis included 383 total posts. Applied thematic analysis using Dedoose was used to identify overarching analytic outputs.

**Results:**

Five major analytic outputs emerged: emotional and psychological responses, communication and information challenges, practical and logistical challenges, family and social dynamics, and connections with forum participants. Emotional responses included feelings of fear and “scanxiety,” alongside positive feelings such as gratitude and resilience facilitated by peer support. Participants frequently reported gaps in healthcare communication and shared strategies for navigating caregiving logistics and financial strain. Family interactions were described as sources of both strength and tension, and forums were described as vital avenues for emotional support, validation, and practical advice.

**Conclusion:**

These findings highlight the unique role of online forums in providing valuable insights into patient and caregiver priorities and show their potential to complement traditional research methods. Future research should explore the long-term impact of forum engagement on mental health and caregiving outcomes to maximize their utility in healthcare settings.

## Introduction

Brain tumors represent a significant healthcare challenge, not only due to their complex pathophysiology but also because of their profound impact on patients and informal caregivers. Individuals living with brain tumors often face a wide array of physical, cognitive, and emotional challenges, such as debilitating fatigue, memory deficits, and mood disturbances, which significantly impair their quality of life [1, 2]. These challenges can lead to a sense of loss of autonomy and identity, as patients adjust to new limitations and uncertainties [3]. Informal caregivers of brain tumor patients—individuals who provide support and are typically unpaid—in turn, shoulder substantial burdens of care, including managing complex medical regimens, navigating healthcare systems, and providing emotional support, all of which contribute to high levels of informal caregiver stress, burnout, and a decline in their own physical and mental health [4–7]. Research has shown that informal caregivers of brain tumor patients often experience higher levels of strain compared to informal caregivers of other chronic conditions due to the rapid progression and unpredictable nature of brain tumors [2, 7]. Understanding the experiences of brain tumor patients and their informal caregivers is critical for guiding patient-centered care and interventions. Traditionally, insights into patient and informal caregiver needs have stemmed from structured clinical studies, qualitative interviews, and survey-based research [5, 6, 8]. However, these methods, while valuable, may fail to capture the full breadth of lived experiences as they are often limited by participant recruitment biases and rigid research frameworks.

With the abundance of online communication platforms, patients and informal caregivers have increasingly turned to discussion forums for sharing personal experiences, seeking information, and finding support [9, 10]. These forums often contain rich, unstructured data that reflects real-time concerns, questions, and coping strategies. The growing reliance on digital platforms for health communication emphasizes their relevance in understanding real-time patient and informal caregiver experiences [11]. Online forums provide a unique, unfiltered lens into discussions, revealing emerging topics and priorities that may not yet be captured in formal research [12]. Such forums offer relative anonymity, and research has shown cancer patients are more willing to disclose personal details about intimate concerns on forums than they are during qualitative interviews [13]. Research among brain tumor patients found online support forums allowed them to share feelings that might have hurt relatives, offered valuable connections with others, and provided a place to share and obtain resource suggestions [14, 15]. These platforms could potentially serve as low-cost, accessible information sources for healthcare providers and researchers to monitor and analyze concerns and, in turn, adapt clinical care to align with the evolving needs of this population. Studies on other chronic conditions, such as diabetes and cancer, or on diseases, in general, have demonstrated that online health forums can provide unique insights into the lived experiences of patients and informal caregivers, often complementing and, in some cases, deviating from what is reported in traditional clinical literature [16–19]. However, there remains a significant gap in the literature regarding the use of online forum data to analyze and understand the specific challenges faced by brain tumor patients and their informal caregivers.

This study aims to explore the content of brain tumor online discussion forums to learn more about the experiences, concerns, and coping strategies shared by brain tumor patients and informal caregivers. Insights learned from this analysis of unfiltered conversations outside of the clinical and research settings can inform researchers and healthcare professionals about lived experiences and lead to more holistic, patient-centered approaches to care and support.

## Methods

This study included individuals who posted or responded in publicly accessible online discussion forums for brain tumor patients and informal caregivers. The forums were selected due to their focus on brain tumors and their established reputations as trusted resources. Posts were authored by patients, caregivers, and, in some cases, moderators. The moderators did not identify themselves as healthcare professionals. The terms and conditions of each forum explained they were post-moderated, meaning user posts were published immediately, and moderators could review comments that violated guidelines after they were posted. Original posts and responses were collected in September 2024. Descriptions of the forums are presented in Table 1. The Institutional Review Board at Northern Illinois University determined the study to be exempt from human subjects review in accordance with federal regulation criteria.

**Table 1:** Public brain cancer forums used with description

Forum name	Description
American Brain Tumor Association [20]	“The American Brain Tumor Association Connections Support Community connects patients, families, friends and caregivers for support and inspiration. This community is sponsored by the American Brain Tumor Association, an Inspire trusted partner.”
American Cancer Society—Cancer Survivors Network—Brain Cancer Forum [19]	“The Cancer Survivors Network is a peer support community for cancer patients, survivors, caregivers, families, and friends! CSN is a safe place to connect with others who share your interests and experiences.”
MacMillan Cancer Support—Brain Cancer Forum [18]	“A brain tumour support group for anyone diagnosed with a brain cancer/tumour and their family members & loved ones. It’s a safe space to find emotional support, discuss treatment and share experiences.”
Mayo Clinic—Brain Tumor Connect [21]	“An online community, connecting patients and family caregivers with each other. You do not have to be a Mayo Clinic patient or caregiver to join the conversations.”

### Data analysis

One researcher (MLF) extracted the first 20 posts and the first six responses in each of the four forums using the passive/unobtrusive data collection method [20] and following the logic of previous research that examined caregiver-related online discussion forum content [16]. This method involves harvesting and analyzing text-based conversational exchanges so researchers can follow participants’ conversations and interactions. Posters’ usernames and names contained in comments were removed for identity protection. These forums did not include any spam posts. Some posts had fewer than six responses. The analysis included 80 initial posts with 303 responses, for a grand total of 383 posts included in the data set.

Using applied thematic analysis, the data were first independently examined by three researchers (CM, SE, and MLF) using Dedoose. They read the original posts and responses collected from each forum and iteratively coded the qualitative data. The four-person author group met to discuss the codes, resolve coding disagreements, and make refinements based on group discussion. They independently constructed analytic outputs from the data then met to collaboratively discuss the analytic outputs and reexamine the data to ensure the constructed analytic outputs appropriately characterized the forums’ contents.

To determine frequency of co-occurrence, we utilized the “code co-occurrence” function within Dedoose. This tool generated a matrix displaying how frequently pairs of codes were applied to the same or overlapping excerpts. By analyzing these co-occurrence patterns, we identified which of our constructed analytic outputs commonly appeared together in participants’ discussions [21]. Additionally, none of the four authors had lived experience as a brain tumor patient, and caregiving experience varied; one researcher had previously cared for a person with a brain tumor.

## Results

This study analyzed four online discussion forums dedicated to brain tumor patients and their informal caregivers: the American Brain Tumor Association, Mayo Clinic, MacMillan Cancer Support, and the American Cancer Society [22–25]. These forums provided information on the challenges individuals face navigating a brain tumor diagnosis, common questions for which they sought answers, and the support they received from online communities. Discussions included a wide range of topics, encompassing personal coping strategies, medical advice, and the profound psychological impact of a brain tumor diagnosis. Table 2 shows the frequency of patient, informal caregiver, and moderator posts and responses.

**Table 2:** Number and author of posts and responses per forum

	Patient	Informal caregiver	Moderator
Mayo Clinic	31	24	12
MacMillan	17	38	20
ACS	36	54	3
ABTA	52	69	8

Note: Responses were excluded from analysis if the poster’s role (i.e., patient, caregiver, or moderator) could not be determined

Additionally, the forums offered data on tumor type, affected brain regions, and treatment approaches (see Table 3). The data showed that chemotherapy, surgery, and radiation were, by far, the most frequently discussed treatments across the forums. Other treatments mentioned were steroids, alternative therapies like wearable device NovoTTF-100L (Optune), the medication bevacizumab (Avastin), rehabilitation, headache and nausea medications, immunotherapies, and seizure medications. Differences emerged in the types of tumors and brain regions discussed, with gliomas being the most frequently mentioned and specific focus on areas like the cerebellum and brain stem varying by forum. Given the wide variability in tumor biology, prognosis, and patient experience, Table 4 highlights the distinct concerns, symptoms, and care challenges associated with each tumor type as reflected in participants'posts. While tumor-specific experiences are noted, the focus of this analysis remains on the broader patterns of patient and caregiver concerns across brain tumor forums.

**Table 3:** Brain tumor and treatment content

	Number of posts
**Brain tumor type**	**207**
Gliomas	119 (57.5%)
Meningioma	32 (15.5%)
Unspecified	21 (10.1%)
Pituitary	13 (6.3%)
Lymphoma	6 (3.0%)
Medulloblastoma	5 (2.4%)
Rare (ependymoma, pineoblastoma, neuroblastoma)	5 (2.4%)
Brain metastases	3 (1.4%)
Schwannoma	3 (1.4%)
**Brain region**	**32**
Temporal lobe	10 (31.3%)
Brain stem	9 (28.1%)
Frontal lobe	5 (15.6%)
Cerebellum	4 (12.5%)
Occipital lobe	3 (9.4%)
Pineal gland	1 (3.1%)
**Treatment type**	**345**
Chemotherapy	91 (26.4%)
Surgery	89 (25.8%)
Radiation	87 (25.2%)
Steroids	21 (6.1%)
Alternative therapies	15 (4.3%)
Avastin	13 (3.8%)
Rehabilitation	9 (2.6%)
Other (headache, nausea medications)	9 (2.6%)
Immunotherapies	7 (2.0%)
Seizure medications	4 (1.2%)

**Table 4:** Brain tumor-specific challenges and themes

Brain tumor type	Top challenges	Common analytic outputs
Gliomas	Rapid decline, seizures, informal caregiver burden, poor coordination of care, palliative support issues	Hopelessness, grief, fragmented care systems
Meningioma	Delayed care, uncertainty, visual and neurological issues, anxiety over watch-and-wait approach	Dismissal by providers, frustration with slow timelines
Unspecified	Nonspecific or vague symptoms, unclear diagnosis, fear of unknown	Emotional isolation, lack of guidance from care teams
Pituitary	Hormonal imbalances, body pain, vision issues, delays in diagnosis, surgical fears	Misdiagnosis, quality of life concerns, relief post-surgery
Lymphoma	Cognitive changes, seizures, long inpatient stays for chemo, need for caregiver advocacy	High treatment burden, executive function changes, family stress
Medulloblastoma	Cognitive/motor effects, radiation side effects in young children, long-term development impact	Parent grief, emotional trauma, long-term survivorship concerns
Rare (ependymoma, pineoblastoma, neuroblastoma)	Post-surgical rehab, motor/speech impairments, long recovery, parental distress	Uncertainty around treatment options, difficulty accessing trials
Brain Metastases	Cognitive decline, dual-disease burden, lack of coordinated care	Rapid changes in function, end-of-life planning, emotional strain
Schwannoma	Facial numbness, hearing loss, dizziness post-radiation, balance issues	Rare side effects, navigating persistent sensory issues

The analysis of code co-occurrence revealed that information-seeking often intersected with discussions about chemotherapy, imaging, surgery, coping strategies, and feelings of fear or nervousness. The term “online forum” frequently occurred alongside information-seeking, coping, receipt or provision of nonprofessional advice, surgery, and glioblastoma multiforme. It’s important to note that patient and informal caregiver reports were largely similar, often addressing the same core topics; however, concerns about care coordination and advocacy were expressed almost exclusively by informal caregivers. Through a comprehensive analysis, five major analytic outputs emerged; we present these analytic outputs below. Table 5 depicts representative quotations from each analytic output.

**Table 5:** Representative quotations from analytic outputs

Analytic output	Participant quotation
Emotional and psychological responses	I have two young children. My husband and I are scared and devastated and I don’t have anyone to talk to who understands how lonely and bitter I feel to have to go through this when everyone around me keeps living normally. (Patient) [22]
	I am struggling to cope with feelings of not being good enough. There are so many lives lost to cancer. There are people who fought harder and longer than myself. There's often the thought in the back of my mind “Why did I survive when so many people do not?” (Patient) [23]
	There are things you can do something about and things you can't. Focus on what you can do and accept what you can't. Focus on the present, what can you do to reassure them that they are loved, that you are there for them? (Informal caregiver) [22]
	I will celebrate my GBM4 6 th anniversary soon, and I am thriving. My mantra is to worry less, love more, and live freely. (Patient) [24]
Communication and information challenges	I don’t know how to bring up these topics with my doctor—the idea of trying to tell the people who know me is even scarier. (Patient) [23]
	At first I was afraid to tell family members about my tumor because what most people know and think about brain tumors is that they're an automatic death sentence. (Patient) [24]
Practical and logistical challenges	[Name] is now 67. Medicare will not pay for rehab and cancer treatments at the same time. It's been 3 months. Very little recovery so far. (Informal caregiver) [23]
	But my daughter is very concerned with things that are really bugging her. She is worried about the utilities, also about transportation for her son when he gets home from Utah. She lost her car because she has been out of work because of this. (Informal caregiver) [23]
	Adult Social Care…said they may be able to put a package together to enable me to go back to work but when they told me that would be a cost of £20.20 an hour on top of wrap around school care it doesn’t make returning to work viable! (Informal caregiver) [22]
Family and social dynamics	My sister devised a system for me to keep all of my medications straight—pill sorters, calendars, and alarms—it made all the difference. (Patient) [23]
	I started a Facebook group that friends and family could join to get updates on my journey. This was very helpful for me. It made me feel like I had a team of supporters following me. (Patient) [23]
	The worry never goes away, I pray a lot and have a lot of family and friend support. (Informal caregiver) [24]
	My family dismisses my symptoms as aging—they don’t see how different this feels to me. (Patient) [24]
	He is pushing me away and I don’t understand why. I feel like my marriage isn't going to survive this. (Informal caregiver) [24]
Connection with forum participants	We are a club none of us wanted to join, but I am so grateful for every member here. (Patient) [24]
	So, I definitely think the medication could be causing some of the problems your husband is experiencing and it's worth asking about. Medications can be helpful but can also cause so many side effects that they can feel worse than the original ailment. Maybe your husband's doctor has another alternative to Keppra that will work better for your husband. (Informal caregiver) [25]

### Analytic Output 1: emotional and psychological responses

A prevalent analytic output across the forums was the emotional rollercoaster experienced by patients and informal caregivers. Common emotions included fear, uncertainty about the future, and “scanxiety” (anxiety related to upcoming medical scans). Patients shared moments of both despair and hope, while caregivers often expressed guilt and frustration. The forums also highlighted positive emotional responses, especially in posts discussing long-term survival and coping. Many participants reported a shift in their perspective, leading to an increased appreciation for life. Others expressed a renewed focus on joy and gratitude, despite their diagnosis. Multiple participants described transitioning from a busy lifestyle to one where they were more present in everyday life.

### Analytic Output 2: communication and information challenges

Many participants detailed their struggles with accessing timely and clear information from healthcare providers. Delays in communication, a lack of second opinions, and confusion over treatment options were recurring concerns. Participants also expressed difficulty in communicating their diagnosis to their loved ones. Participants frequently posed questions about tumor types, treatment protocols, and prognosis. Many participants noted that healthcare systems often left them feeling “lost in the process,” turning to forums to understand complex medical jargon and navigate their care. In particular, discussions about interpreting imaging results and advocating for more detailed explanations from providers were common. Participants also frequently mentioned the value of second opinions and encouraged fellow forum participants to seek them as a means of empowerment.

### Analytic Output 3: practical and logistical challenges

Practical challenges were prominently discussed, including financial strain, managing medications, and arranging caregiving logistics. Medicare coverage, power of attorney, and access to palliative care were notable concerns. Participants shared advice on managing medication side effects and balancing caregiving responsibilities with personal well-being. Financial barriers were a recurring issue, with some participants discussing the challenges of affording specialized treatments or traveling for care. Strategies for organizing caregiving tasks, such as creating shared calendars or enlisting extended family members, were frequently shared. The forums also served as a venue for discussing the pros and cons of hospice and palliative care, with participants offering their perspectives on when and how to make these decisions.

### Analytic Output 4: family and social dynamics

Family and friend relationships played a dual role as sources of strength and stress. Patients expressed gratitude and appreciation for their loved ones’ support. In some instances, caregivers reported a strong support network that helped them get through challenging times. Participants also described negative interactions within their families. Some posters focused on family disbelief or denial, while others focused on the lack of support. Informal caregivers often reported minimal caregiving assistance from their extended families or reported disagreement regarding caregiving duties or their loved one’s medical care. Many also discussed the challenges associated with juggling multiple priorities simultaneously, while some described how the diagnosis had put a major strain on their relationships.

### Analytic Output 5: connection with forum participants

The majority of participants expressed a strong connection to others in the discussion forum and described the forum as a “lifeline.” Mentions of gratitude for these communities were notably more frequent than expressions of fear or despair. Participants turned to the forums for emotional support and practical advice and to provide and receive encouragement. When medical questions were posed, participants either offered medical advice, encouraged discussions with doctors, or provided resources related to their question.

## Discussion

The findings from this study offer valuable insights into the experiences of brain tumor patients and their caregivers through the lens of online discussion forums. The major five analytic outputs identified—emotional and psychological responses, communication and information challenges, practical and logistical challenges, family and social dynamics, and connection with forum participants—align with the broader literature on chronic illness and caregiver burden and highlight the unique and multifaceted challenges associated with brain tumors [7, 26].

The emotional and psychological responses observed in this study, such as “scanxiety” and coping with uncertainty, are consistent with previous studies on the psychological toll of brain tumors [27, 28]. However, our study showed a new dimension—that of transitioning from negative feelings to positive feelings after interacting with other forum participants. The shift from fear and anxieties to gratitude and appreciation indicated a reframing of their situation through shared experiences with other community members. This finding emphasizes the therapeutic value of storytelling in health communication, a concept well-documented in narrative medicine literature [29].

The communication and information challenges identified highlight a persistent gap in patient and provider communication, particularly regarding prognosis, treatment options, and interpreting complex medical information. Existing research has shown that inadequate communication can exacerbate patient anxiety and reduce trust in healthcare systems [30]. When patients and caregivers are well-informed, they are better equipped to make healthcare decisions [8]. The forums’ role in bridging these gaps suggests that healthcare providers might consider integrating similar peer-support models into their practice to provide more comprehensive care and support.

Practical and logistical challenges, such as financial strain and caregiving logistics, reflect systemic issues within healthcare access and affordability, particularly with chronic and terminal illnesses. These findings are consistent with studies emphasizing the economic and emotional burden on caregivers [31, 32]. Patients also face significant practical challenges including navigating complex treatment regimens, managing symptoms, and maintaining quality of life amidst these burdens [33]. The strategies shared within the forums, from medication management tips to navigating hospice care, demonstrate the potential for practical patient or caregiver-driven solutions to complement formal healthcare resources.

Family and social dynamics, including both the supportive and stressful aspects of caregiving relationships, mimic analytic outputs from current caregiver literature, highlighting the dual role of family as a source of both stress and strength [1, 34]. The forums’ capacity to provide an outlet for informal caregivers to share their experiences and receive validation from others is a crucial finding, suggesting that online discussion forums may support improved resilience among caregivers [35, 36]. Online discussion forums also have the potential to help informal caregiver groups with specific relationships to the care recipient, such as widowed caregivers who often face particular challenges, such as letting go of the caregiver role and experiencing grief from the loss of one’s life partner [37].

Chemotherapy, surgery, and radiation were prominent topics in the discussion boards, illustrating their importance in treating brain tumors. However, this high frequency also demonstrates that they are a significant source of fear, anxiety, and uncertainty among participants. Participants often sought information from other community members on these topics, which also highlights a potential gap in provider communication and education. Steroid treatment was also a frequent area of concern; this is not surprising, given its great benefit but also high potential for severe physical, physiological, and psychological side effects [38]. Participants discussed rehabilitation at a lower rate. The fewer rehabilitation posts may indicate that it is less an area of concern or that it is underutilized in this population. This underutilization is consistent with previous research that has shown that individuals with brain tumors receive less rehabilitation therapy compared with individuals with stroke and traumatic brain injury, despite similar physical and cognitive deficits [39].

The importance of connecting with other forum participants emerged as a central analytic output in this study, reflecting the unique role of online communities in providing emotional and informational support to patients and caregivers. Previous research has established the value of peer support in reducing caregiver isolation and distress [40]. However, our findings extend this understanding by emphasizing the potential of these interactions. Patients and caregivers in this study described a shift from feelings of loneliness and helplessness to empowerment and validation after engaging with others in similar situations. This process allowed participants to process their caregiving experiences through shared stories and mutual encouragement [25]. These connections with others, often described as lifelines during times of overwhelming stress, align with previous literature emphasizing the psychological benefits of storytelling as a strategy to foster resilience and emotional well-being [41].

It is important to note that there are potential dangers in participants accepting advice from other forum users as medical fact. While these forums can offer important emotional support and shared experience, it is essential that patients and caregivers ultimately discuss major health decisions with their medical team to ensure accuracy and safety. These online spaces should be viewed as complementary to, and not replacements for, professional medical guidance. Additionally, healthcare providers can play a proactive role by being present, either personally or professionally, on such forums to help prevent misinformation [42].

Our analysis indicated similar types of posts and responses for patients and informal caregivers, with just care coordination being expressed more with informal caregivers than patients, which is consistent with previous research [43]. This difference between patient and caregiver posts illustrates the need for healthcare systems to develop targeted strategies that address caregivers’ unique challenges in coordinating care, potentially through enhanced communication channels and support services. Furthermore, considering the diverse nature of brain tumors, it may be beneficial for future research to examine tumor-specific forums to better understand the distinct needs of patients and caregivers associated with different tumor types. Such focused studies could provide more insights into the specific challenges and support mechanisms relevant to each subgroup, thereby informing more tailored interventions and resources.

This study had some limitations. Participant and caregiver data was self-reported, which may lead to self-selection bias and recall bias. Those who chose to engage in the discussion forums may not accurately represent the wider population of brain tumor patients and caregivers. Further, they may not accurately remember the details of their treatment or experience, which could impact the veracity of their descriptions. Another limitation is that the forums were based in the USA and the UK, and most participants were likely from these countries. This impacts generalizability, as brain tumor patients and caregivers in other health care systems may face different experiences. The forums were also publicly available, which may have limited what a poster would share.

The results of this study have several implications for healthcare practice and policy. First, healthcare providers should recognize the value of online forums as complementary resources for patient and caregiver education. Integrating moderated, evidence-based forums into clinical practice could enhance patient satisfaction and self-efficacy. Second, online forums provide unfiltered data to providers, allowing them to stay current with the most significant caregiver and patient stressors. It is important to note that such data must first be interpreted and contextualized. This could occur through qualitative analysis like in our study or using advanced analytical tools like machine learning algorithms [44]. Third, addressing the systemic barriers discussed in the forum, such as financial strain and access to timely care, will require policy-level interventions, such as expanded Medicare coverage for advanced treatments and informal caregiver support programs.

Future research should build on these findings by examining the long-term impact of participation in online forums on patient and caregiver outcomes. For example, examining active engagement in online forums and its relation to improved mental health, reduced caregiver burden, increased self-efficacy, or better healthcare decision-making may be beneficial. Additionally, qualitative studies could explore the experiences of underrepresented groups within these forums, such as patients from diverse socioeconomic backgrounds, to ensure that digital health solutions are equitable and inclusive. By addressing these areas, future research can help harness the full capabilities of online forums as valuable supplements to traditional health care.

## Data Availability

The datasets generated and analyzed during this study are available from the corresponding author on reasonable request.
